# Prevention of colitis-associated cancer by selective targeting of immunoproteasome subunit LMP7

**DOI:** 10.18632/oncotarget.14579

**Published:** 2017-01-10

**Authors:** Niyati Vachharajani, Thorsten Joeris, Maik Luu, Sabrina Hartmann, Sabine Pautz, Elena Jenike, Georgios Pantazis, Immo Prinz, Markus J. Hofer, Ulrich Steinhoff, Alexander Visekruna

**Affiliations:** ^1^ Institute for Medical Microbiology and Hygiene, Philipps University of Marburg, Marburg, Germany; ^2^ Section of Immunology and Vaccinology, National Veterinary Institute, Technical University of Denmark, Frederiksberg, Denmark; ^3^ Department of Neuropathology, Philipps University of Marburg, Marburg, Germany; ^4^ Institute of Immunology, Hannover Medical School, Hannover, Germany; ^5^ School of Life and Environmental Sciences, The University of Sydney, New South Wales, Australia

**Keywords:** colon cancer, inflammation, immunoproteasome, NF-κB

## Abstract

Chronic inflammation is a well-known risk factor in development of intestinal tumorigenesis, although the exact mechanisms underlying development of colitis-associated cancer (CAC) still remain obscure. The activity and function of immunoproteasome has been extensively analyzed in the context of inflammation and infectious diseases. Here, we show that the proteasomal immunosubunit LMP7 plays an essential role in development of CAC. Mice devoid of LMP7 were resistant to chronic inflammation and formation of neoplasia, and developed virtually no tumors after AOM/DSS treatment. Our data reveal that LMP7 deficiency resulted in reduced expression of pro-tumorigenic chemokines CXCL1, CXCL2 and CXCL3 as well as adhesion molecule VCAM-1. As a consequence, an impaired recruitment and activity of tumor-infiltrating leukocytes resulting in decreased secretion of cytokines IL-6 and TNF-α was observed. Further, the deletion or pharmacological inhibition of LMP7 and consequent blockade of NF-κB abrogated the production of IL-17A, which possesses a strong carcinogenic activity in the gut. Moreover, *in vivo* administration of the selective LMP7 inhibitor ONX-0914 led to a marked reduction of tumor numbers in wild-type (WT) mice. Collectively, we identified the immunoproteasome as a crucial mediator of inflammation-driven neoplasia highlighting a novel potential therapeutic approach to limit colonic tumorigenesis.

## INTRODUCTION

Individuals suffering from inflammatory bowel disease (IBD) face a high risk of developing colorectal cancer [1]. Environmental and genetic factors play a crucial role in the onset of inflammation-driven carcinogenesis whereby the loss of function of tumor suppressors such as adenomatous polyposis coli (APC) and increased activation of β-catenin are mostly accompanied by increased expression of pro-tumorigenic cytokines such as IL-6, TNF-α and IL-17A. Intestinal epithelial cells expressing high levels of TNFR1 are highly sensitive to TNF-α, which is able to induce NF-κB-mediated oncogenic pathways [2]. Similar to IL-6 and IL-17A, the new member of the IL-6 cytokine family, IL-11, profoundly activates epithelial STAT3 signalling [3]. Accordingly, in the murine experimental model of colitis-associated cancer (CAC), both the tumor multiplicity and growth were diminished in the absence of IL-17A, IL-6, IL-11 or TNF-α [4]. Taken together, the hyperactive intestinal immune system provides external factors for intestinal epithelium contributing to its constitutive NF-κB and STAT3 activation, which enhances the cross-talk of intra-tumoral signaling, resulting in excessive proliferation and resistance to apoptosis.

The constitutive proteasome, which is essential for disposal of damaged proteins and maintenance of protein turnover in eukaryotic cells, comprises three catalytic subunits, β1, β2, and β5. In mammals, the exposure of cells to type I and II interferons leads to the formation of immunoproteasome that can be distinguished from its constitutive homologue through *de novo* synthesis and assembly of the catalytic immunosubunits LMP2 (β1i), MECL-1 (β2i), and LMP7 (β5i) [5]. Simultaneously with induction of immunoproteasome, IFN-γ upregulates the expression of other factors which are needed for efficient antigen presentation such as transporter associated with antigen processing (TAP) and proteasome activator 28 (PA28) [6]. The primary function of immunoproteasome has been connected to the optimal generation of peptides for MHC class I presentation [7]. Recent reports have revealed other important roles of immunoproteasomes in immune system. During inflammation, immunoproteasomes help constitutive proteasomes to handle the enhanced pool of proteasomal substrates preventing aggregate formation of damaged proteins in the cells [8]. Importantly, we and others have shown in mouse models of colitis and rheumatoid arthritis that immunoproteasomes were essential for initiation of inflammatory processes [9–11].

With regard to the activation of classical NF-κB signalling pathway, contradictory data have been published in earlier reports. While some researcher groups do not see any impairment of NF-κB activity in the absence of intact immunoproteasome, we and others have observed defective NF-κB activation in mice devoid of LMP7 or LMP2 [10, 12, 13]. Given the broad functional aspects of its activities in immune cells, we hypothesised that the immunoproteasome might be a crucial factor involved in the onset of inflammation-driven carcinogenesis.

## RESULTS

### Reduction of colitis-associated carcinogenesis in the absence of LMP7

**Figure 1:**
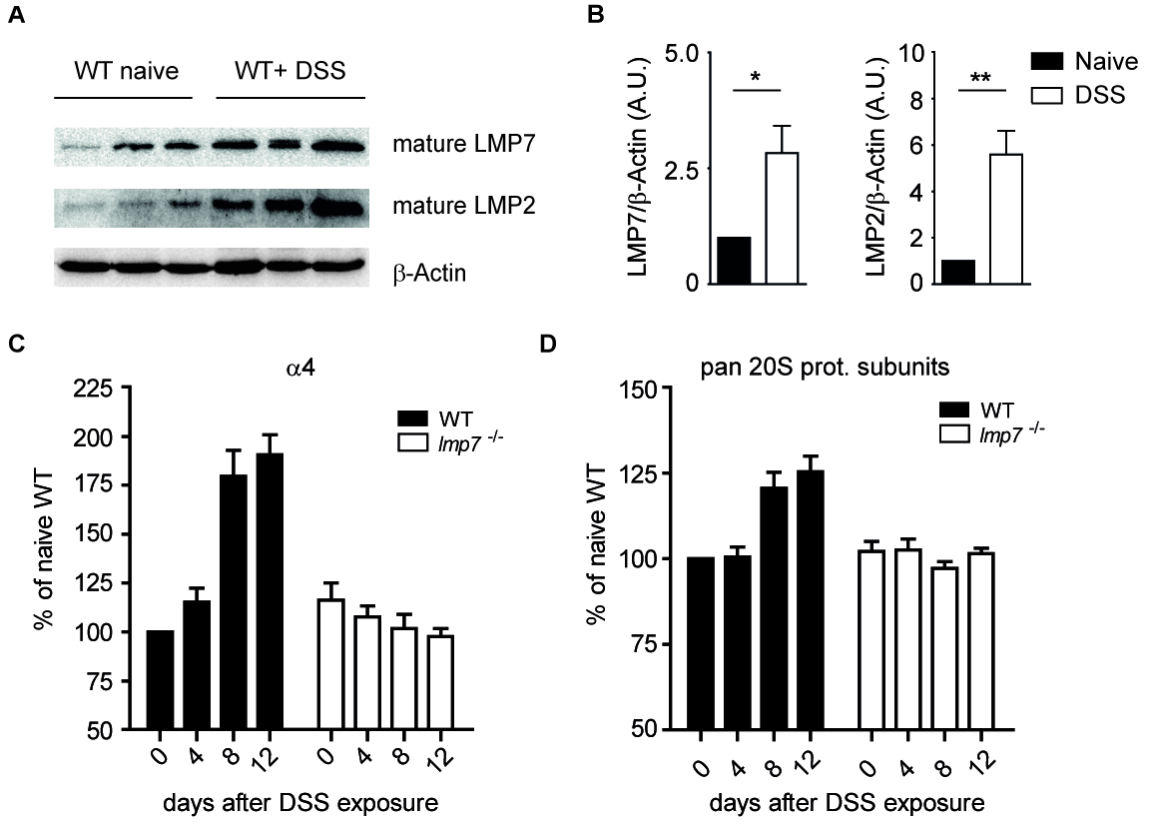
Abundance of proteasomal subunits after treatment of WT mice with DSS **A.** Immunoblot analysis for LMP7 and LMP2 in naïve and DSS-treated mice (day 8 after induction of colitis). β-Actin served as a loading control. A representative of two independent experiments is shown. **B.** Densitometric quantification of immunoblot analyses for LMP2 and LMP7 expression on day 8 after DSS treatment of WT mice. **C** and **D.** Densitometric analysis for protein expression of structural proteasomal subunit α4 and for all pan-20S subunits (MP3 Ab) on indicated days after exposure to 3% DSS. The membranes were stained against GAPDH as loading control. Data represent mean ± SEM. Three independent experiments were performed.

The immunoproteasome is crucial for the optimization of CD8^+^ T cell-mediated immune responses during viral or bacterial infections [14]. Novel data have demonstrated that immunoproteasomes also efficiently control the proinflammatory activity of immune cells [10, 13]. In contrast to lymphoid tissues with high expression of immunoproteasomes, the colonic lamina propria exhibits low amounts of this enzymatic complex [15]. Particularly, the expression of LMP2 and LMP7 is much lower than that of their counterparts β1 and β5, respectively (Supplementary Figure 1), which might be one important mechanism to protect the host from immune overreaction to commensal antigens. Previously, we and others have shown that the mice devoid of intact immunoproteasomes display reduced colonic inflammation and tissue destruction [9, 11]. During the induction of colitis, we observed increased expression of immunoproteasome subunits LMP2 and LMP7 in the inflamed colon of DSS-treated WT mice (Figure 1A and 1B). Recently, we reported an up-regulation of proteasome quantity in WT but not in LMP7 deficient mice infected with *Listeria monocytogenes* [16]. Comparable to this finding, we detected a significant increase in the expression of α4 subunit, which is a structural part of both constitutive and immunproteasomes, on day 8 after induction of colitis by dextran sodium sulfate (DSS) in WT mice. On the contrary, no up-regulation of this proteasomal subunit was observed in DSS-treated *lmp7*^−/−^ animals. Moreover, by using a pan-20S proteasome antibody, an increase of about 25 % of total amount of 20S proteasomes was found in WT mice after exposure to DSS for 8 and 12 days. However, no increase in overall abundance of mature 20S proteasome was seen in *lmp7*^−/−^ mice during colonic inflammation (Figure 1C and 1D). Thus, it seems that LMP7 deficient mice are unable to efficiently upregulate expression and assembly of proteasomal proteins in response to ongoing colonic inflammation.

**Figure 2:**
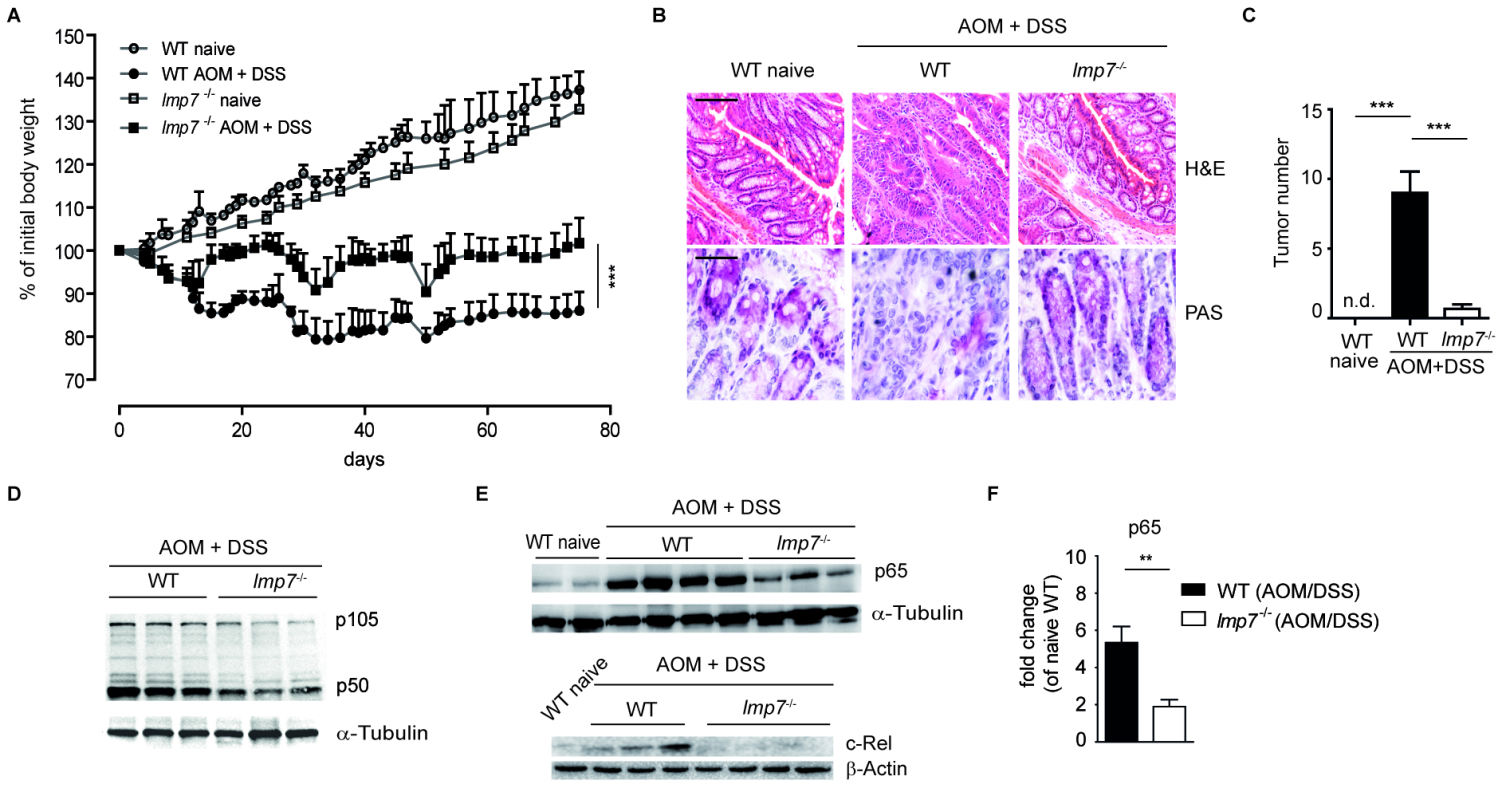
Immunoproteasome subunit LMP7 is essential for development of colitis-associated cancer (CAC) **A.** CAC was induced in WT and *lmp7*^−/−^ mice and the change in weight was monitored in naïve and AOM/DSS-treated mice over a period of 75 days. Data represent mean ± SEM (n=10-12 mice per group). ***P<0.001. **B.** Representative images of H&E- and PAS-stained colon sections of control or AOM/DSS-treated mice (day 80). Scale bars: 100µm for H&E and 200µm for PAS staining. **C.** Colonic tumor incidence in naïve and AOM/DSS treated mice (day 80). Data represent mean ± SEM (n=10-12 mice per group). ***P<0.001. **D** and **E.** Reduced NF-κB levels in LMP7 deficient mice after induction of CAC. Immunoblot analysis for p105/p50 (D), p65 and c-Rel (E) was performed for the whole colonic tissue of AOM/DSS-treated mice (day 80). α-Tubulin or β-Actin served as a loading control. A representative of two experiments is shown. **F.** Densitometric analysis for colonic p65 expression on day 80 after induction of CAC in WT and *lmp7*^−/−^ mice. Data represent mean ± SEM of two independent experiments and are normalized to p65 expression in the colon of naïve WT mice. **P<0.01.

We speculated that the lower proteasome quantity observed in animals devoid of LMP7 might also influence the development of colitis-associated cancer (CAC). To investigate the role of immunoproteasomes in development of inflammation-driven carcinogenesis, WT and *lmp7*^−/−^ mice were treated with azoxymethane (AOM)/DSS and induction of colitis-associated neoplasia in colon was monitored for 80 days. During the course of AOM treatment and cyclic administration of DSS in drinking water, a profound body weight loss accompanied with occurrence of multiple tumors was observed in WT mice, whereas less significant loss of body weight, no visible adenocarcinomatous lesions and no loss of mucous-producing goblet cells were detectable in H&E or PAS-stained colonic tissue sections of LMP7 deficient animals (Figure 2A-2C). Overall, these data suggest that the inflammatory responses driven by immunoproteasomes are involved in colonic tumorigenesis.

The decrease in proteasome amount and altered catalytic activity in the absence of LMP7 might have a significant impact on many cellular signalling cascades. The involvement of transcription factor NF-κB in both inflammatory cells and colonic epithelium has been shown to play a crucial role in colitis-associated tumorigenesis [3, 17–19]. Our previous analysis has clearly shown that the activation of canonical NF-κB pathways was significantly impaired in the absence of immunoproteasomal subunits [13]. Following the exposure of colonic cells to pathogen-associated molecular patterns (PAMPs) and pro-inflammatory cytokines due to the damage caused by DSS, the proteasomes in colonic epithelial and immune cells rapidly process NF-κB precursor protein p105 and degrade IκBα. In accordance with decreased total proteasome abundance in LMP7 deficient cells, we observed strong reduction in the expression of NF-κB proteins detected by lower p105, p50, p65 and c-Rel levels in the colon of LMP7 deficient animals as compared to WT mice 80 days after initiation of CAC (Figure 2D-2F).

### The impaired recruitment of tumor-associated leukocytes into the colon of LMP7 deficient mice

**Figure 3:**
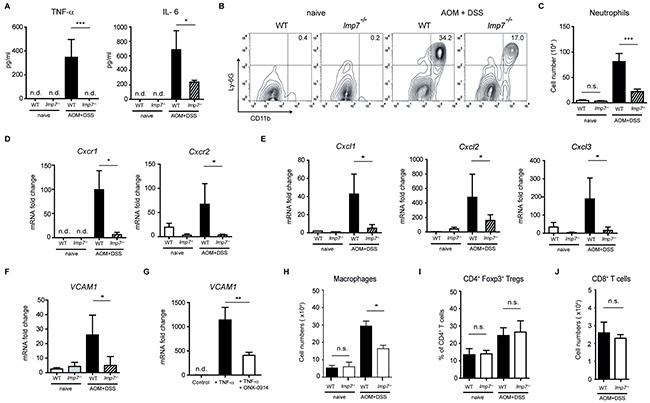
Regulation of neutrophil-recruiting chemokines and VCAM-1 by LMP7 during progression of CAC **A.** Secretion of TNF-α and IL-6 by *ex vivo* colon explants of naïve or AOM/DSS-treated mice at day 30 after induction of CAC was measured by ELISA. Data represent mean ± SEM (n = 10-12 mice per group). **B** and **C.** Leukocytes were isolated from the colon of naïve and AOM/DSS-treated mice on day 30 after CAC induction. Cells were stained and gated on SSC^high^ granulocyte gate. Frequency (B) and total cell numbers (C) of CD11b^+^Ly-6G^+^ neutrophils were analysed by flow cytometry. **D-F.** Quantitative real-time PCR analyses for expression of *Cxcr1*, *Cxcr2* (D) *Cxcl1*, *Cxcl2*, *Cxcl3* (E) and *VCAM1* (F) was performed using colon tissues from naïve and AOM/DSS-treated mice at day 30 after CAC induction. **G.** *VCAM1* expression in HUVECs treated with TNF-α (10 ng/ml) in the presence or absence of ONX-0914 (100 nM). Pooled data from two independent experiments are shown. **H.** Absolute cell numbers of colonic MHC-II^+^ CD11b^+^ macrophages on day 30 after CAC induction. **I**. Colonic Treg frequencies on day 30 after AOM/DSS treatment of WT and LMP7 deficient mice. **J**. Cell numbers of CD8^+^ T cells in colon of WT and LMP7 deficient mice on day 30 after CAC induction. For the analyses (C-F and H-J), data represent mean ± SEM (n =10-12 mice per group). For all experimental analyses: n.d., not detectable, n.s., not significant, *P<0.05, **P<0.01, ***P<0.001.

An inflammatory environment promotes cancer development by providing newly emerging tumors with factors essential for their growth, angiogenesis and metastasis. Novel data have identified TNF-α, IL-17A and IL-6 as crucial link between enhanced activity of immune system and colon carcinogenesis [20]. To explore the relevance of immunoproteasome in the regulation of pro-tumorigenic cytokines, we measured inflammation-driven TNF-α and IL-6 secretion after initiation of AOM/DSS treatment. Colonic explants of AOM/DSS-treated LMP7 deficient mice secreted almost negligible amounts of these cytokines as compared to strong production in colon of WT animals (Figure 3A). Colitis and carcinogenesis are intimately linked by enhanced activity of infiltrating immune cells and increased secretion of chemokines and cytokines. Recently, it has been shown that recruitment of neutrophils to the inflamed colon favours development of inflammation-driven tumorigenesis [21, 22]. Furthermore, the genes encoding chemokines, namely *Cxcl1* and *Cxcl2*, which are responsible for recruitment of CXCR2^+^ blood neutrophils, are strongly up-regulated in colonic adenocarcinomatous lesions [23]. Accordingly, blocking CXCR2 or CXCL1 profoundly suppressed intestinal inflammation-driven carcinogenesis [23, 24]. On day 30 after induction of CAC, we detected a significantly reduced influx of neutrophils in the colon of *lmp7*^−/−^ mice in comparison to WT animals (Figure 3B and 3C). Moreover, the relative mRNA expression of *Cxcl1*, *Cxcl2* and *Cxcl3* as well as that of their receptors *Cxcr1* and *Cxcr2* was significantly reduced in the colon of AOM/DSS-treated LMP7 deficient mice as compared to WT mice (Figure 3D and 3E). The migration of neutrophils into the peritoneal cavity during induction of peritonitis has been described to be mediated via CXCL1 and CXCL2 [25]. To test if observed defective expression of *Cxcl1* and *Cxcl2* in *lmp7*^−/−^ mice has an impact on recruitment of inflammatory cells during peritonitis, we measured the percentage of neutrophils in peritoneal lavages 4 h after intraperitoneal injection of thioglycollate. Similar to the detected migratory defects in intestine, the absence of LMP7 significantly reduced the influx of neutrophils in the peritoneum (Supplementary Figure 2).

Transmigration of neutrophils through endothelial cells is mediated via activation of specific adhesion receptors such as vascular cell adhesion molecule-1 (VCAM-1). In contrast to WT counterparts, almost no up-regulation of this molecule during induction of CAC in the colon of *lmp7*^−/−^ mice was detected, which suggests that several mechanisms contribute to observed migratory defects (Figure 3F). Previously, it was shown that TNF-α-triggered VCAM-1 expression in endothelial cells is under transcriptional control of NF-κB and that the blockade of NF-κB activity by unspecific proteasome inhibitor MG132 was able to suppress the expression of VCAM-1 and *in vitro* transmigration of neutrophils [26, 27]. To examine if *VCAM1* expression is directly regulated by LMP7, we stimulated human umbilical vein endothelial cells (HUVECs) with TNF-α in the presence or absence of specific LMP7 inhibitor ONX-0914. These data revealed that the inhibition of the immunoproteasome subunit LMP7 downregulates *VCAM1* gene expression in endothelial cells (Figure 3G). Importantly, the expression of *Cxcl1*, *Cxcl2* and *Cxcl3* is also transcriptionally regulated by canonical NF-κB signaling pathway [28]. For *Cxcl1*, *Cxcl2*, *Cxcl3* and *VCAM1*, we were able to find several NF-κB binding sequences in proximal promoter regions of humans, rats and mice. Particularly, the regulatory region 100-200 base pairs upstream of transcription start site (TSS) comprised the typical NF-κB binding motif with highly conserved sequence in all examined genes and species (Supplementary Table 1). Therefore, we concluded that, in *lmp7*^−/−^ mice, the reduced NF-κB activity might be responsible for impaired expression of *VCAM1*, *Cxcl1*, *Cxcl2* and *Cxcl3*. Further, we analysed immune cell populations that play an anti- or pro-tumorigenic role in development of CAC. While Foxp3^+^ Tregs and CD8^+^ T cells were comparable in WT and LMP7 deficient animals after AOM/DSS treatment, a moderate defect in macrophage cell numbers was detected in the colonic lamina propria of LMP7 deficient mice (Figure 3H-3J). Taken together, during the inflammation-associated-carcinogenesis, the immunoproteasome subunit LMP7 is crucially involved in the regulation of expression of pro-tumorigenic chemokines, cytokines and adhesion molecules.

### Colonic IL-17A production is reduced in LMP7 deficient mice during colitis-associated carcinogenesis

**Figure 4:**
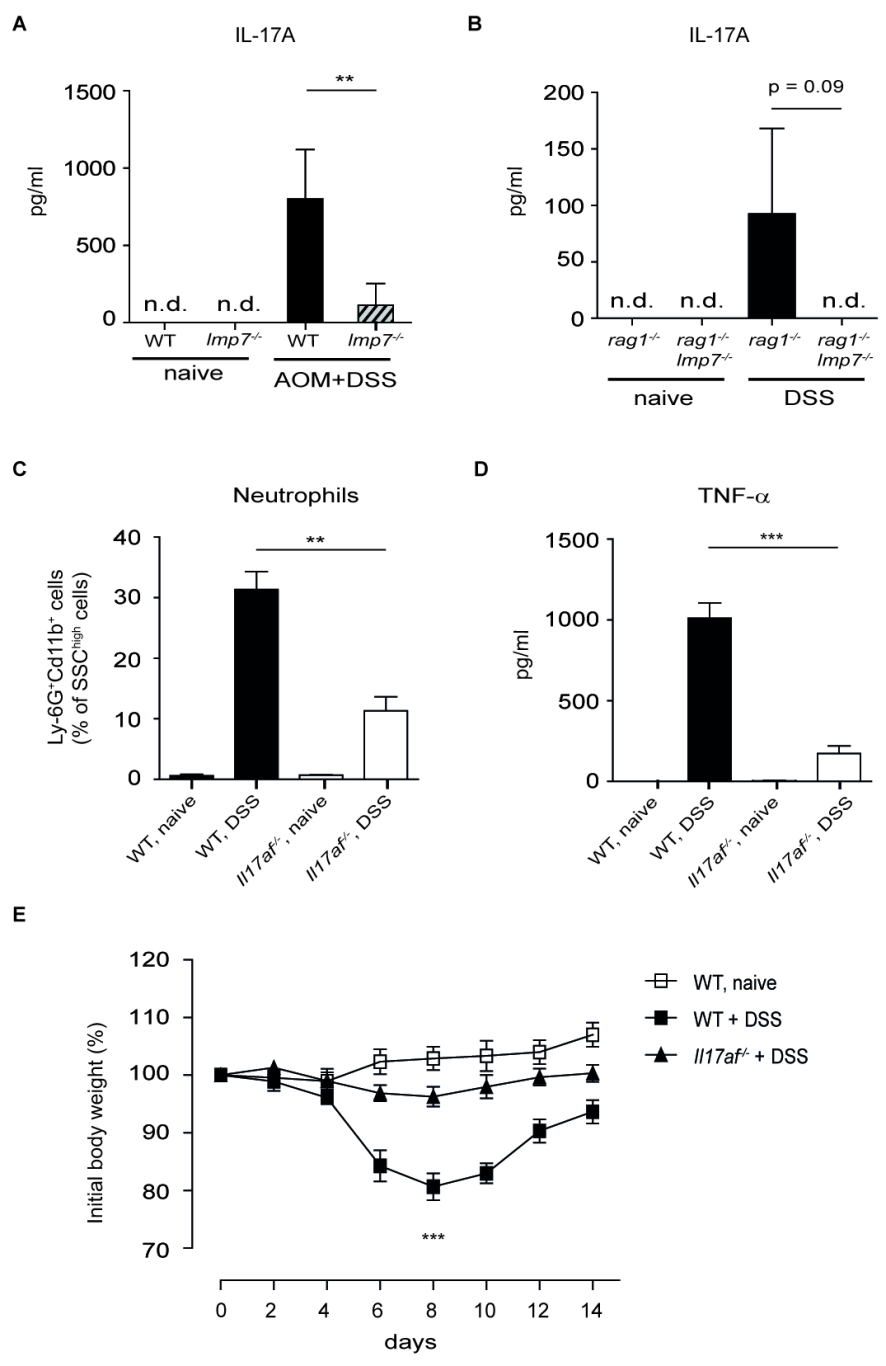
Deletion of LMP7 leads to reduction of IL-17A production **A.** Secretion of IL-17A by *ex vivo* colon explants of naïve or AOM/DSS-treated mice at day 30 after induction of CAC was measured by ELISA. Data represent mean ± SEM (n = 10-12 mice per group). **B.** IL-17A secretion in overnight colon cultures at day 8 after exposure of *rag1*^−/−^ and *rag1^−/−^lmp7*^−/−^ mice to 2.5 % DSS. Data represent mean ± SEM (n = 8 mice per group). **C.** Total numbers of colonic CD11b^+^Ly-6G^+^ neutrophils at day 6 after induction of colitis was analysed by flow cytometry. **D.** Secretion of TNF-α in overnight colon cultures at day 6 after exposure of WT and *il17af*^−/−^ mice to 2.5 % DSS. **E.** Monitoring of weight loss in *il17af*^−/−^ mice after oral treatment with 2.5 % DSS for 5 days. For (C-E), data represent mean ± SEM (n =8 mice per group), **P<0.01, ***P<0.001.

In an experimental CAC model, IL-17A deficient mice have been shown to exhibit significantly lower tumor numbers compared to WT mice [29]. A recent study has demonstrated a redundant role for adaptive and innate sources of IL-17A production during the colonic carcinogenesis indicating the need for therapeutic targeting of cytokine itself rather than cellular origin of IL-17A [30]. During the development of colitis or CAC, we observed a significantly reduced secretion of IL-17A in the colon of *lmp7*^−/−^ and *rag1*^−/−^*lmp7*^−/−^ mice as compared to WT and *rag1*^−/−^ control animals, respectively (Figure 4A and 4B). These data suggest that the enzymatic activity of LMP7 regulates IL-17A production not only in Th17 cells but also in innate lymphoid cells, and possibly in other cell types. Interestingly, during intestinal inflammation, a very similar phenotype was observed between *lmp7*^−/−^ and *il17af*^−/−^ mice, which displayed less weight loss, lower frequencies of neutrophils and decreased colonic secretion of TNF-α as compared to WT mice (Figure 4C-4E).

**Figure 5:**
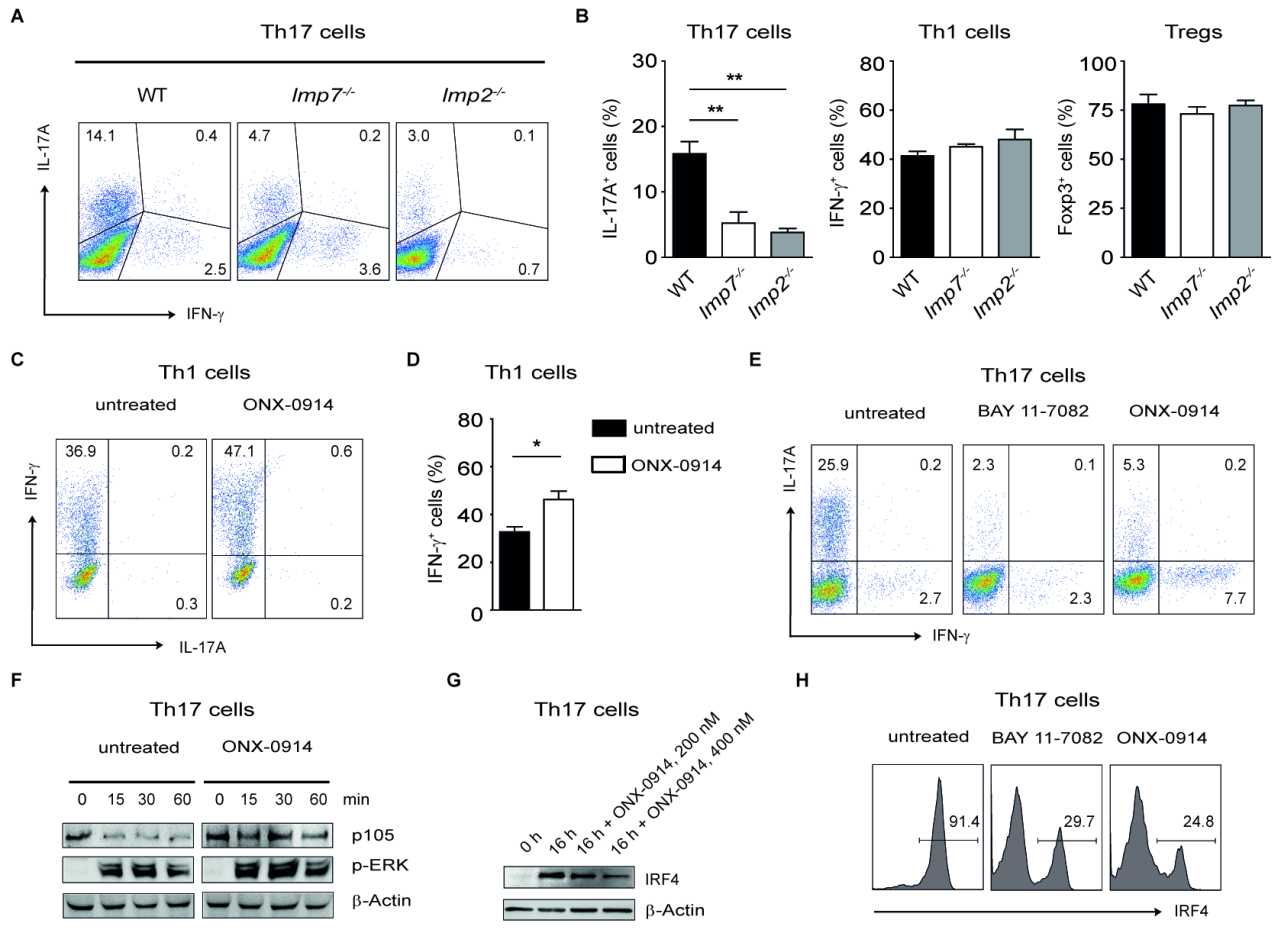
Specific blockade of LMP7 reduces Th17- but not Th1-mediated immune responses **A.** Representative dot plots showing the expression of IL-17A and IFN-γ in purified CD4^+^ T cells cultured under Th17 conditions for three days. Three similar experiments were performed. **B.** CD4^+^ T cells were purified from WT, *lmp7^−/−^* and *lmp2^−/−^* mice and cultured under Th17-, Th1- and Treg-inducing conditions for three days, respectively. Three similar experiments were performed. Data are shown as mean ± SEM. **P<0.01, ***P<0.001. **C** and **D.** CD4^+^ T cells were purified from spleens and lymph nodes of WT mice and cultured with or without ONX-0914 (250 nM) under Th1-inducing conditions. Representative dot plots show the expression of IFN-γ and IL-17A (C). Data from two similar experiments (D) are displayed as mean ± SEM. *P<0.05. **E**. Representative FACS dot plots showing the IL-17A and IFN-γ expression in CD4^+^ T cells cultured under Th17 conditions for three days in the presence of inhibitors BAY 11-7082 and ONX-0914 (both 300 nM), respectively. Three experiments were performed. **F**. Analysis of NF-κB and ERK activation in CD4^+^ T cells after stimulation with PMA/ionomycin for indicated times. One group of cells was additionally treated with ONX-0914 (400 nM). Two similar experiments were performed. **G**. Immunoblot analysis of IRF4 expression in Th17 cells cultured for 16 h in the presence of ONX-0914. **H**. Th17 cells were cultured for three days with or without BAY 11-7082 and ONX-0914 (both 300 nM) and subsequently intracellular staining for IRF4 was performed. Three similar experiments were performed.

Previously, it has been published that the blockade of LMP7 activity by its selective inhibitor ONX-0914 reduced expression of IFN-γ and IL-17A by Th1 and Th17 cells and increased Foxp3 expression by Tregs, respectively [31]. To determine whether *lmp7*^−/−^ and *lmp2*^−/−^ T cells exhibit defective effector functions, we isolated CD4^+^ T cells from WT animals and mice deficient for LMP2 or LMP7 and cultivated them for three days under Th1-, Th17- and Treg-inducing conditions. In contrast to previous study, we were able to observe only selective defect under Th17-polarizing conditions. There were no significant differences in frequencies of IFN-γ^+^ Th1 cells or Foxp3^+^Tregs between WT, LMP2 and LMP7 deficient mice. In contrast, IL-17A expression was impaired in *lmp7*^−/−^ and *lmp2*^−/−^ Th17 cells as compared to WT counterparts suggesting a direct role for immunoproteasomes in controlling production of this pro-tumorigenic cytokine (Figure 5A and 5B, and Supplementary Figure 3). Similarly, we were not able to detect defective IFN-γ expression and Th1 differentiation after treatment of WT CD4^+^ T cells with ONX-0914. In contrary, we even observed a slight increase in production of IFN-γ upon LMP7 blockade (Figure 5C and 5D). Finally, Th17 cells treated with a specific NF-κB inhibitor BAY 11-7082 or LMP7 inhibitor ONX-0914 showed defective expression of IL-17A compared with untreated Th17 cells (Figure 5E). We and others have recently shown that NF-κB signalling regulates the induction of IRF4 in the lymphocytes, which is required for IL-17A production by Th17 cells [32–34]. Following the T cell receptor (TCR) activation, the IKK-induced p105 degradation by proteasome is needed to release the associated NF-κB subunits to translocate into the nucleus and modulate the gene expression. Here, we demonstrate that ONX-0914-treated Th17 cells were not capable of activating NF-κB p105 signalling pathway and of inducing IRF4 expression, whereas the phosphorylation of ERK was not affected (Figure 5F-5H). Similarly, IRF4 levels were reduced in Th17 cells treated with BAY 11-7082 as compared with untreated Th17 cells (Figure 5H). These results indicate non-redundant role of LMP7/NF-κB/IRF4 axis in regulation the expression of IL-17A by Th17 cells.

### Selective inhibition of LMP7 suppresses inflammation-driven colon cancer

**Figure 6:**
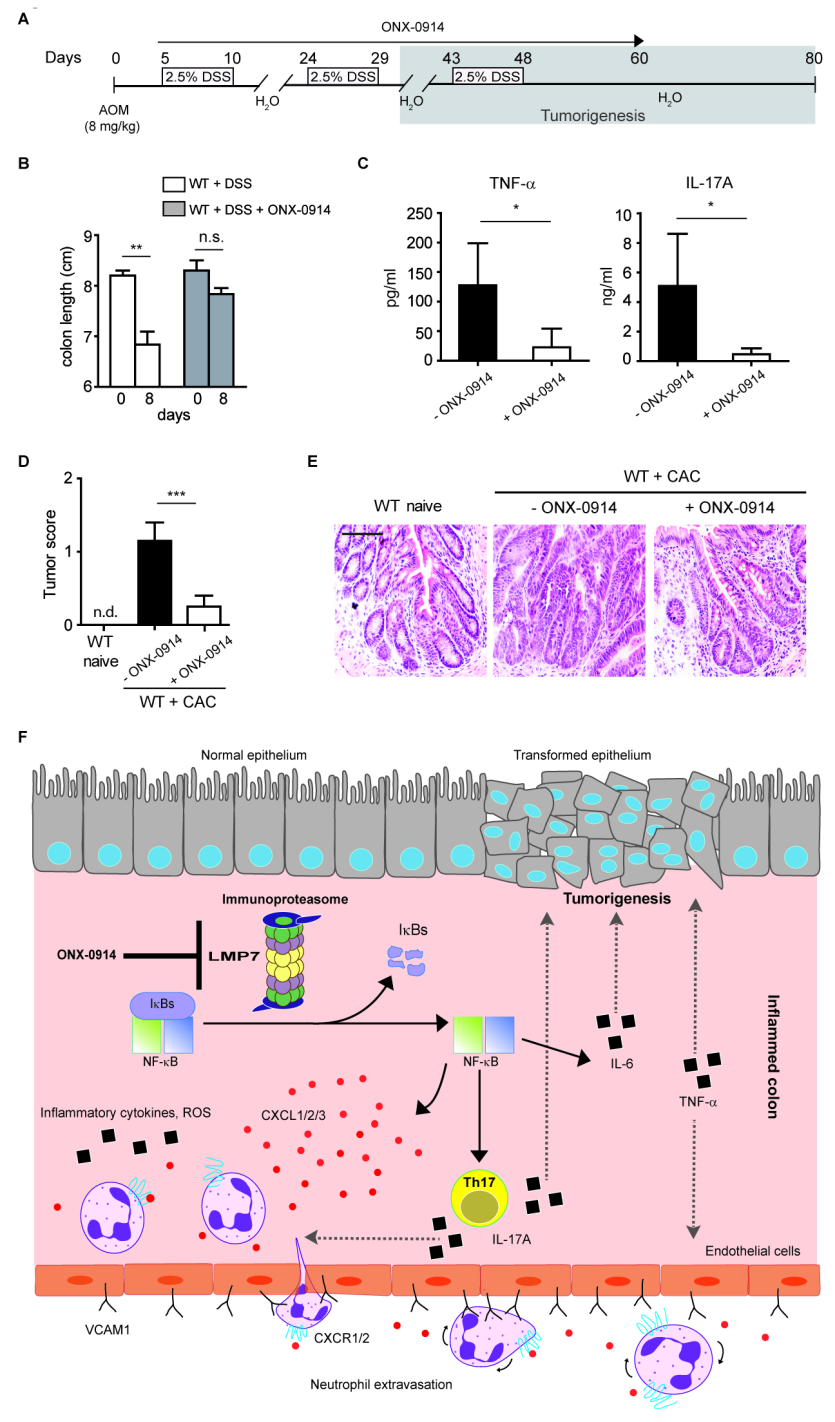
Specific blockade of LMP7 inhibits development of CAC **A.** Scheme for the experimental course for induction of CAC in WT mice using 8 mg/kg AOM and 3 cycles of DSS (2.5%). Mice were treated with 6 mg/kg ONX-0914 three times per week starting at day 5 after AOM administration. **B.** Colon length of DSS-treated WT mice with or without ONX-0914 (6 mg/kg mice, i.p. administration daily) was measured at day 8 after colitis induction (n=6 mice per group). Data represent mean ± SEM, **P<0.01, n.s., not significant. **C**. Cytokine secretion by colon *ex vivo* explants of AOM/DSS treated WT mice in the presence or absence of ONX-0914 was measured by ELISA at day 30 after CAC induction. *P<0.05. **D.** Colonic tumor score in naïve and AOM/DSS-treated WT mice with or without ONX-0914 administration (day 80 after CAC induction). For (C and D), data represent mean ± SEM and are representative of two independent experiments (n =10-12 mice per group), n.d., not detectable, ***P<0.001. **E.** Representative images of H&E-stained colon sections of naïve and AOM/DSS-treated WT mice injected with ONX-0914 as described in (A). Histology analysis was performed at day 80 after CAC induction. Scale bar: 100µm. **F.** Schematic overview showing the role of LMP7 in development of CAC. In inflamed colon, the increased expression of immunoproteasome subunit LMP7 leads to NF-κB/IRF4-mediated secretion of IL-17A which impacts the recruitment of neutrophils into the colon and promotes carcinogenesis. Additionally, LMP7/NF-κB axis regulates the expression of chemokines CXCL1-3 and adhesion molecule VCAM-1. The inhibition of LMP7 with ONX-0914 affects the tumor formation by dampening the pro-inflammatory mediators.

The LMP7 inhibitor ONX-0914 has been shown to attenuate the progression of inflammation in experimental models of arthritis and colitis [9, 10]. As the *VCAM1* gene expression and IL-17A production was also down-regulated after the treatment of cells with ONX-0914, we hypothesised that proteosomal immunosubunits might be a specific target for development of anti-inflammatory therapies needed for dampening the ongoing inflammation-driven cancer. A recent study has described high selectivity of ONX-0914 for LMP7 both *in vitro* and *in vivo*, however, the treatment of mice with higher doses of this inhibitor was shown to be very toxic and even lethal [10]. We tested the dose-dependent toxicity of ONX-0914 and found that administration of 6 mg ONX-0914 per kg mice at every second day starting at day 5 after induction of CAC was well tolerated and had a significant impact on the course of carcinogenesis. The treatment of WT mice with ONX-0914 led to less pronounced shortening of colon length and reduced production of pro-tumorigenic cytokines TNF-α and IL-17A in the colon after administering AOM and DSS or DSS alone (Figure 6A-6C). Although some hyper-proliferation of colonic epithelial cells was observed after ONX-0914 administration, mice treated with this inhibitor had markedly reduced tumor numbers and size in comparison to normal WT animals as shown in representative H&E sections, macroscopic tumor count and microscopic tumor progression score (Figure 6D and 6E).

In summary, our data identify immunoproteasome as a crucial factor contributing to initiation and progression of inflammation-driven tumorigenesis. Our novel findings show that secretion of pro-tumorigenic cytokines such as IL-17A as well as of chemokines is controlled by immunoproteasome subunit LMP7. The LMP7-dependent up-regulation of CXCL1, CXCL2, CXCL3 and VCAM-1 expression leads to alternations in the microenvironment of tumors and progression of colorectal carcinogenesis (Figure 6F). Given that colon cancers often arise from chronic inflammation and selective inhibition of immunoproteasome subunit LMP7 results in decreased production of pro-inflammatory and pro-tumorigenic molecules, this study provides a therapeutic rationale for targeting immunoproteasomes in colorectal carcinogenesis associated with colitis.

## DISCUSSION

Diverse cellular functions of proteasomes have been described, whereby the regulation of cell cycle, apoptosis, cellular proliferation and activation of transcription of various genes are closely connected to development of cancer [35]. The ubiquitin-proteasome system (UPS) is considered as a promising target for cancer therapy and some proteasome inhibitors such as bortezomib are already implemented in the treatment of malignant diseases [36]. Mice with a targeted deletion of the immunoproteasome subunit LMP7 had originally been described to have reduced levels of MHC class I cell surface expression [37], which might lead to the conclusion that cancer immunosurveillance and CTL-mediated antitumor immunity might be impaired in these animals. On the contrary, given the crucial role of immunoproteasomes in regulation of various pro-inflammatory mediators, one might also speculate on a potential pro-carcinogenic role for immunoproteasomes during the progression of chronic inflammation. In accordance, we found that genetic ablation or therapeutic inhibition of LMP7 reduced pro-inflammatory responses and subsequent CAC development in the AOM/DSS model of carcinogenesis.

Chronic inflammation appears to be one of the most frequent risk factors for development of intestinal tumor growth and progression to malignancy [4]. Recent studies have highlighted the mechanisms by which the cytokines secreted by inflammatory cells and regulated by the transcription factor NF-κB such as TNF-α, IL-6 and IL-17A stimulate tumor development and progression [20]. Although NF-κB is constitutively activated in many tumors, mutations in the family of NF-κB transcription factors are very rare. Thus, during the colon carcinogenesis, the pro-tumorigenic function of NF-κB might originate from mutations in regulatory molecules in inflammatory cells acting upstream of this transcription factor. In the mouse model of CAC, the intense NF-κB-mediated interaction between tumors and infiltrating immune cells is reflected in the observation that myeloid-specific deletion of NF-κB reduces tumor burden in mice [38].

Our novel data demonstrate that the immunoproteasome subunit LMP7 is crucially involved in exaggerated colonic inflammatory reactions that favour development of intestinal carcinogenesis. In the AOM/DSS model of colon carcinogenesis, we observed reduced NF-κB activity and significantly less tumors in LMP7 deficient mice as compared to WT animals. The previous studies investigating kinetics of IκBα degradation and NF-κB activation pointed to the dysregulated NF-κB signaling pathway in the absence of LMP7 and LMP2 [8, 12, 39]. The observed delay in the nuclear translocation of active NF-κB subunits might be explained by altered catalytic specificity and decreased total proteasome amount in the absence of immunoproteasomes. These defects highlight an essential function for immunoproteasomes in immune cells apart from their role in processing of MHC class I antigens.

Our result indicate that the immunoproteasome function was associated with a variety of inflammatory processes in the gut such as recruitment of neutrophils and regulation of expression of chemokines, cytokines and adhesion molecules. Interestingly, we observed a defective production of IL-17A in innate and adaptive immune cell compartments in the absence of LMP7. This is consistent with novel data suggesting that both innate and adaptive cellular sources of IL-17A contribute to progression of colon carcinogenesis [30]. It seems that pro-tumorigenic Th17 cells are crucially dependent on ubiquitin-proteasome pathway and especially on the expression of LMP7 to activate NF-κB/IRF4 axis needed for intact production of IL-17A.

Recently, efforts have been made to develop immunoproteasome-specific inhibitors since many unspecific proteasome inhibitors are associated with unwanted and toxic effects in spite of their promising therapeutic applications. A number of immunoproteasome-specific inhibitors are currently being tested in pre-clinical and clinical studies and among them ONX-0914 has been proven as a potent agent to treat inflammatory disorders such as colitis and rheumatoid arthritis [10, 31]. This inhibitor has been shown to be 20- to 40-fold more selective for LMP7 subunit over its constitutive counterpart β5 [10]. Notably, the treatment of WT mice with ONX-0914 reduced expression of pro-tumorigenic cytokines and results in significantly attenuated symptoms of experimental CAC. Moreover, LMP7 blockade by ONX-0914 was associated with impaired expression of pro-tumorigenic cytokine IL-17A and adhesion molecule VCAM-1 *in vitro*. Although we did not observe reduced frequency of intestinal CD8^+^ T cells in AOM/DSS treated mice lacking LMP7, a treatment with LMP7 inhibitor might have some undesired side effects such as increased susceptibility to certain infections and altered antigen processing of tumor neoantigens. Together, these observations identify immunoproteasome, in particular its subunit LMP7, as a potential therapeutic target for treatment of colon carcinogenesis.

## MATERIALS AND METHODS

### Mice

C57BL/6 WT mice were purchased from Charles River Laboratory. *lmp7*^−/−^, *lmp2*^−/−^, *il17af*^−/−^, *rag1*^−/−^ and *rag1*^−/−^ *lmp7*^−/−^ mice (on C57BL/6 background) were bred at the animal facility of the Biomedical Research Center, University of Marburg. *il17af*^−/−^ mice were obtained from Immo Prinz, Institute of Immunology, Hannover Medical School, Hannover, Germany.

### Tumor and colitis induction

Sex-matched mice were injected with AOM (Sigma-Aldrich) intraperitoneally (i.p.) at a dose of 8 mg/kg body weight. After 5 days, mice were treated with 2.5% DSS (MP Biomedicals) administered via drinking water for 5 days, followed by 14 days of normal drinking water. The DSS cycle was repeated twice as indicated. Mice were sacrificed on day 80 for analyses. In some experiments, acute colitis was induced by treating mice orally with 2.5 % DSS and analysis was performed on day 6 or day 8 after induction of colitis. In some experiments, WT mice were treated with 6 mg/kg of LMP7 inhibitor ONX-0914 (i.p., three times per week) starting at day 5 after AOM administration.

### Histology

Colon tissue cryosections were stained with hematoxylin and eosin (H&E) and periodic acid-Schiff (PAS). Slides were examined blindly by two investigators with bright field microscopy. Digital images were taken using Leica DFC480 camera and Leica Application Suite V3.8 software.

### Isolation of colonic lamina propria mononuclear cells (LPMCs)

Colon pieces were shaken for 30 min at 37°C at 150 rpm in RPMI medium supplemented with 10% FCS, 1 mM L-glutamine and Penicillin/Streptomycin. The epithelial cell fraction was released by vortexing vigorously in PBS (2 % FCS). The remaining colon tissue was digested with 0.4 mg ml^-1^ collagenase D (Roche) and collagenase VIII (Sigma) with constant shaking at 150 rpm for 45 min at 37°C. The cell suspension were washed and resuspended in 40% percoll (Merck). Cells were carefully layered on 70% percoll. After centrifugation at 2000 rpm for 30 min, the LPMCs were collected from the interphase, washed and resuspended in 1ml colon medium. LPMCs were stained with anti-CD11b (clone M1/70, eBioscience), anti-Ly-6G (clone 1A8, BioLegend), anti-CD4 (clone RM4-5, BioLegend), anti-CD8 (clone 53-6.7) and anti-MHC II (clone TIB120, purified at Max-Planck-Institute). Foxp3-expressing Tregs were detected by using the Foxp3 staining kit (clone FJK-16s, eBioscience). Flow cytometry was performed on a FACSCalibur flow cytometer (Becton Dickinson) and the data was analysed using FlowJo 7.6 (Tree Star).

### Colon *ex vivo* explant culture and ELISA

1 cm sections of the proximal colon were washed with PBS to remove feces, and then cut longitudinally. The colon sections were incubated in 1 ml of RPMI medium supplemented with 10% FCS (Sigma) and Penicillin/Streptomycin (PAA). The sections were cultured at 37 °C, 5% CO_2_. Supernatants were harvested after 24 h and cytokine concentrations were determined by ELISA. TNF-α, IL-17A and IL-6 ELISA were performed according to manufacturer's instructions using OptEIA ELISA kits (BD Bioscience).

### Western blot

Using a homogenizer (Ultra-Turrax IKA), pieces of whole colon tissues were lysed in cold RIPA lysis buffer (Sigma) containing protease inhibitors (Thermo Scientific), Na_3_VO_4_ (0.2 mM), NaF (20 mM) with incubation on ice for 15 min and intermittent vortexing. Total protein quantification of the samples was performed with BCA assay kit (Pierce). 20 μg aliquots of protein were separated by electrophoresis in 12% SDS PAGE gels (Bio-Rad) followed by transfer onto a PVDF membrane (Roche). Membranes were blocked for 1 hour, followed by incubating the membrane with primary antibody. Following primary antibodies were used: anti-p65 (Santa Cruz Biotechnology), anti-p105/p50 (eBioscience), anti-LMP7 and anti-LMP2 (Cell Signaling and Santa Cruz Biotechnology, respectively), anti-p-ERK (Cell Signaling) and anti-IRF4 (Santa Cruz Biotechnology). The analysed proteins were detected by chemiluminescence (Biostep) using ImmunoCruz (Santa Cruz Biotechnology). For detection of 20S proteasome subunits, the quantitative two-colour fluorescent immunoblot analysis was performed as described previously [16].

### CD4^+^ T cell differentiation

CD4^+^ T cells from WT, *lmp7*^−/−^ or *lmp2*^−/−^ mice were isolated from spleen and lymph nodes by negative magnetic cell sorting (MACS, Miltenyi Biotec). CD4^+^ T cells were cultured containing plate-bound 5 μg/ml anti-CD3 (clone 145-2C11) and 1 μg/ml soluble anti-CD28 (clone 37.51). For Th17 differentiation, cultures were supplemented with 5 μg/ml α-IFN-γ (clone XMG1.2), α-IL-4 (10% culture supernatants of clone 11B11), 50 U/ml rhIL-2 (Novartis), 1ng/ml rhTGF-β1 (PeproTech) and 40 ng/ml IL-6 (PeproTech). 200-400 nM ONX-0914 (Onyx Pharmaceuticals) or 300-400 nM BAY 11-7082 (Sigma-Aldrich) were added to the Th17 differentiation medium for indicated time points. Cells were restimulated with 750 ng/ml of ionomycin, 50 ng/ml of PMA in presence of 10 µg/ml Brefeldin A (all three substances, Sigma-Aldrich) and were analysed for IL-17A (clone eBio17B7, eBioscience) and IFN-γ (clone XMG1-2, eBioscience) production by intracellular staining (ICS). In some experiments, expression of transcription factor IRF4 (clone 3E4, eBioscience) was analysed by ICS.

### Cell line experiments

Human umbilical vein endothelial cells (HUVECs) (PromoCell) were cultured in endothelial cell growth medium (PromoCell) at 37 °C, 5% CO_2_ in 75 cm^2^ tissue culture flasks. HUVECs from passage 3 to 5 were used for experiments. 1 × 10^5^ cells were seeded in 6-well plates and were allowed to adhere overnight. Cells in the control group were left untreated. For stimulation experiments, HUVECs were pretreated with or without 100 nM ONX-0914 (Onyx Pharmaceuticals) for 2 h. Both untreated and ONX-0914 treated cells were then further stimulated with 10 ng/ml recombinant human TNF-α (PeproTech) and then harvested after 6 h.

### Quantitative real-time PCR

Using TRI Reagent (Sigma-Aldrich), the colon tissues were homogenized and total RNA was extracted according to manufacturer's instructions. RevertAid First Strand cDNASynthesis Kit (Thermo Scientific) was used to generate complementary DNA (cDNA) according to the manufacturer's instructions. Quantitative real-time PCR was conducted on a StepOne Plus device (Applied Biosystems). Quantification of cDNA was carried out by normalization to expression of housekeeping genes HPRT-1 or GAPDH using the ΔΔCt method. The primers used for q RT-PCR are listed in Supplementary Table 2.

### Statistics

Data are presented as mean ± SEM and were analyzed with GraphPad Prism (GraphPad Software). Statistical analyses was done by either Student *t*-test or 1-way ANOVA.

## SUPPLEMENTARY MATERIALS FIGURES AND TABLE


