# Supplementary material for: Prevention of colitis-associated cancer by selective targeting of immunoproteasome subunit LMP7
Source: Oncotarget. 2017 Jan 10;8(31):50447–59. doi: 10.18632/oncotarget.14579 (PMC5584149; doi:10.18632/oncotarget.14579)
Supplement: Supplementary file 1 [file oncotarget-08-50447-s001.pdf]

Prevention of colitis-associated cancer by selective targeting of immunoproteasome subunit LMP7

SUPPLEMENTARY METHODS

Proteasome purification and 2D gel electrophoresis

For the separation of all 20S proteasome subunits by 2D gel electrophoresis, the purification of 20S proteasomes was prepared according to the standard protocol. In brief, proteasomes were purified from colonic lamina propria of C57BL/6 mice using ammonium sulfate precipitation and separation by ultracentrifugation (40,000 rpm, 16 h) on sucrose gradient. Finally, proteasomes were extracted by *fast protein liquid chromatography (*FPLC) and combined 20S proteasome fractions were tested for the purity using 2D gel electrophoresis method.

Induction of peritonitis

In WT and *lmp7*$^{-/-}$ mice, peritonitis was induced by a single i.p. injection of 1.4 ml of 3% sterile thioglycollate (Roth). After 4 hours, mice were sacrificed and cells were recovered by peritoneal lavage by flushing 10ml of cold RPMI medium. The cell suspension was filtered using a 100µm filter and centrifuged at 1500 rpm for 5 min at 4°C. The pellets were resuspended in 1 ml RPMI medium and were immediately stained with anti-Ly-6G and anti-CD11b antibodies. The cells were analysed using flow cytometry.

SUPPLEMENTARY FIGURES AND TABLE

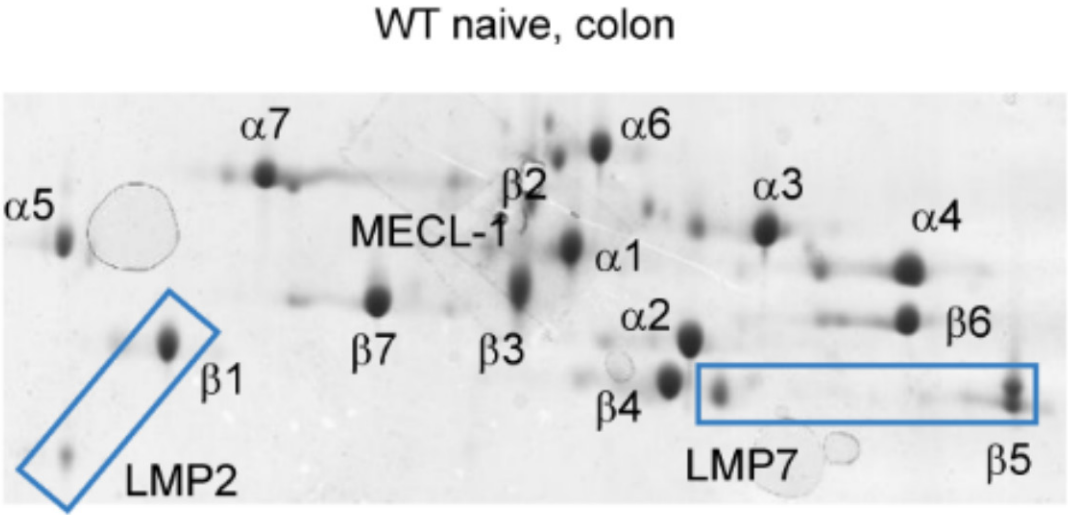

Supplementary Figure S1: 2D-gel displaying basal levels of the proteasomal subunits in murine colon. A representative of three similar experiments is shown.

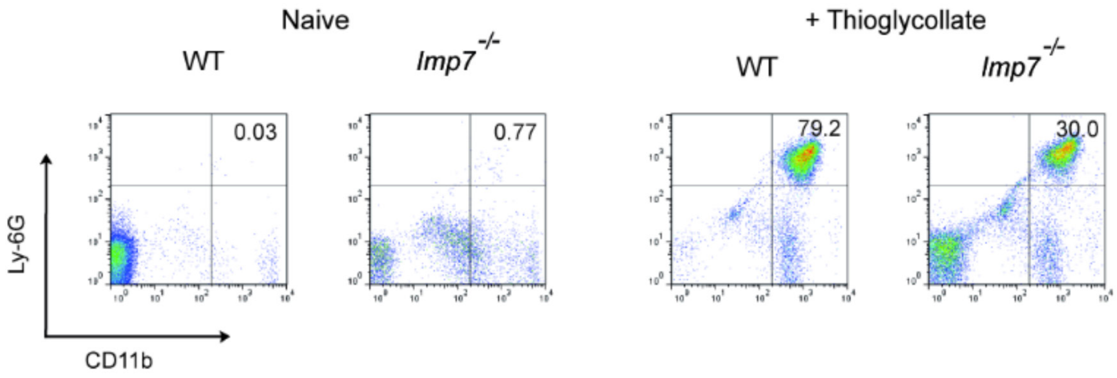

Supplementary Figure S2: Peritoneal exudates from untreated and thioglycollate-treated mice stained for CD11b$^+$ Ly-6G$^+$ neutrophils were analyzed by flow cytometry. A representative of two experiments is shown (n=6 mice per group).

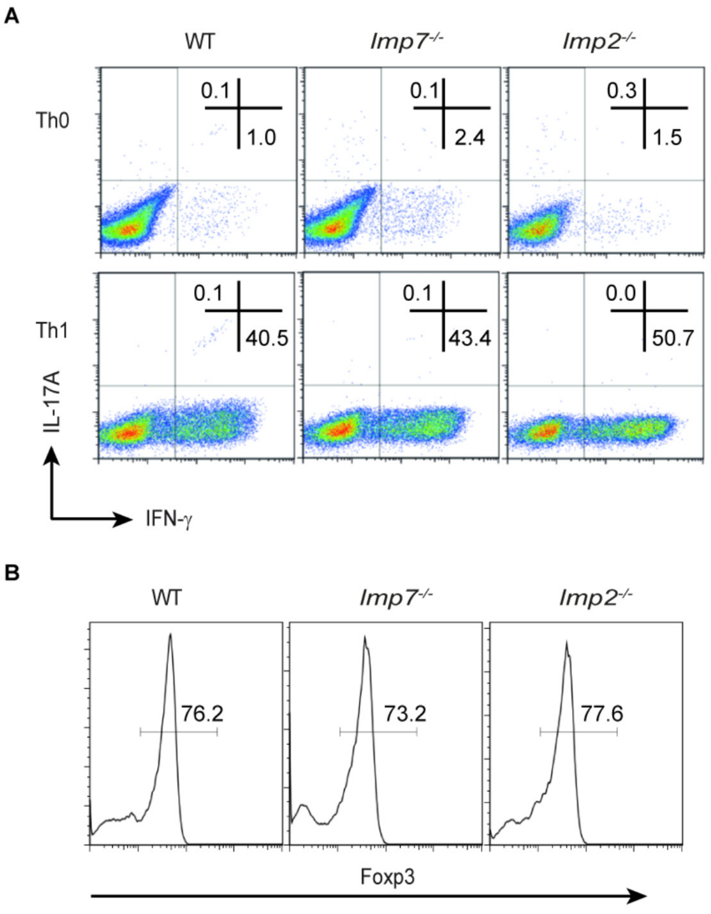

Supplementary Figure S3: A. CD4$^+$ T lymphocytes were purified from spleens and lymph nodes of WT, *Imp7*$^{-/-}$ and *Imp2*$^{-/-}$ mice. Cells were cultured under Th1-inducing conditions or were left unpolarised (Th0) for three days. Representative FACS dot plots show the expression of IFN-γ and IL-17A under Th0 and Th1 conditions. **B.** CD4$^+$ T cells were cultured under Treg-inducing conditions for three days. The histograms display percentages of Foxp3. Three similar experiments were performed.

Supplemental Table S1: Predicted κB binding sites in proximal gene promoters of *Cxcl1*, *Cxcl2*, *Cxcl3* and *VCAM1*

Mus musculus							
Cxcl1	**Pos.**	***Cxcl2***	**Pos.**	***Cxcl3***	**Pos.**	***VCAM1***	**Pos.**
GGGAATTTCCC	-126	GGGCTTTTCC	-114	GGGAATTTCCC	-132	GGGATTTCCC	-141
GGGAATTTCCC	-658	GGGAATTTCCC	-132	GGGTAGGGAA	-1065	GGGAATTCAG	-1428
GGACTTTCC	-746	GGAAGTTCC	-731				
Rattus norvegicus							
Cxcl1	**Pos.**	***Cxcl2***	**Pos.**	***Cxcl3***	**Pos.**	***VCAM1***	**Pos.**
GGGAATTTCCC	-124	GGGCTTTTCC	-114	GGGAATTTCCC	-133	GGGATTTCCC	-140
GGACTTTCC	-748	GGGGATTTCCC	-133			GGATTTTCC	-512
GGAAGTTCCC	-1431	GGAAGTTCCC	-431			GGATTTTTCA	-1487
Homo sapiens							
Cxcl1	**Pos.**	***Cxcl2***	**Pos.**	***Cxcl3***	**Pos.**	***VCAM1***	**Pos.**
GGGCTTTCC	-122	GGGCTTTCC	-113	GGGCTTTCC	-122	GGGATTTCCC	-171
GGGAATTTCCC	-146	GGGAATTTCCC	-142	GGGAATTTCCC	-146		
GAAAGTTCC	-869						

For each gene across three different species (mouse, rat, human), AliBaba2 algorithm was used to predict NF-κB binding sequences within the region 1.5 kb upstream of transcription start site (TSS).
